# The stem cell-associated transcription co-factor, ZNF521, interacts with GLI1 and GLI2 and enhances the activity of the *Sonic hedgehog* pathway

**DOI:** 10.1038/s41419-019-1946-x

**Published:** 2019-09-26

**Authors:** Stefania Scicchitano, Marco Giordano, Valeria Lucchino, Ylenia Montalcini, Emanuela Chiarella, Annamaria Aloisio, Bruna Codispoti, Pietro Zoppoli, Valentina Melocchi, Fabrizio Bianchi, Enrico De Smaele, Maria Mesuraca, Giovanni Morrone, Heather M. Bond

**Affiliations:** 1 Laboratory of Molecular Haematopoiesis and Stem Cell Biology, Department of Experimental and Clinical Medicine, University Magna Græcia, 88100 Catanzaro, Italy; 2 Unit of Gynecological Oncology Research, European Institute of Oncology IRCCS, Via G. Ripamonti 435, 20141 Milano, Italy; 3 German Center for Neurodegenerative Diseases (DZNE), 53127 Bonn, Germany; 4 Tecnologica Research Institute-Marrelli Hospital, 88900 Crotone, Italy; 5 Laboratory of Pre-clinical and Translational Research, IRCCS-CROB, Referral Cancer Center of Basilicata, Rionero in Vulture, Italy; 6 Fondazione IRCCS – Casa Sollievo della Sofferenza, Laboratory of Cancer Biomarkers, San Giovanni Rotondo, 71013 (FG) Italy; 7 Department of Experimental Medicine, University La Sapienza, 00161 Rome, Italy

**Keywords:** Biochemistry, Transcription factors

## Abstract

ZNF521 is a transcription co-factor with recognized regulatory functions in haematopoietic, osteo-adipogenic and neural progenitor cells. Among its diverse activities, ZNF521 has been implicated in the regulation of medulloblastoma (MB) cells, where the Hedgehog (*HH*) pathway, has a key role in the development of normal cerebellum and of a substantial fraction of MBs. Here a functional cross-talk is shown for ZNF521 with the *HH* pathway, where it interacts with GLI1 and GLI2, the major HH transcriptional effectors and enhances the activity of *HH* signalling. In particular, ZNF521 cooperates with GLI1 and GLI2 in the transcriptional activation of GLI (glioma-associated transcription factor)-responsive promoters. This synergism is dependent on the presence of the N-terminal, NuRD-binding motif in ZNF521, and is sensitive to HDAC (histone deacetylase) and GLI inhibitors. Taken together, these results highlight the role of ZNF521, and its interaction with the NuRD complex, in determining the *HH* response at the level of transcription. This may be of particular relevance in HH-driven diseases, especially regarding the MBs belonging to the *SHH* (sonic HH) subgroup where a high expression of ZNF521 is correlated with that of *HH* pathway components.

## Introduction

The Hedgehog (*HH*) pathway is a master regulator of developmental processes whose dysregulation has been linked to a variety of cancers, including those arising from the cerebellum, skin, pancreas, prostate and lung^1,2^. This pathway is activated by the binding of HH ligands to the 12-transmembrane domain protein Patched1 (*PTCH1*), which relieves the repression of smoothened (SMO) and induces its migration to the primary cilium. This results in the dissociation of the suppressor of fused (SUFU)-glioma-associated transcription factor (GLI) complex, allowing the GLI proteins to migrate into the nucleus and act on transcriptional targets, which control cell growth, survival and differentiation^3–6^.

The GLI factors (GLI1, GLI2 and GLI3) represent the major transcriptional effectors of *HH* signalling. These proteins share conserved homology of their zinc finger domains and bind to a consensus motif (GACCACCCA) in the promoters of target genes^7^. It is generally considered that GLI1 acts exclusively as an activator (GLI1A), while GLI2 and GLI3 can act either as activators (GLI2A, GLI3A) or repressors (GLI2R, GLI3R). The combination of activating and repressive forms of the GLI proteins, which act in concert with different signalling pathways in addition to that of *HH*, has led to the concept of a “*GLI* code”, where multiple integrated signals contribute to the control of cell fate^3,8^.

A considerable wealth of information has been accumulated on the activity of the *SHH* (sonic HH) pathway and of the GLI factors, and on their interactions with other intracellular signalling networks. These data are derived from a variety of cases where the imbalance of one pathway will perturb other signalling mechanisms, thus modulating the control of cellular functions. These include, for example, the RAS-MEK-AKT cascade, the *EGF* pathway, signalling by TGFB-SMADS and the *WNT* pathway^1–3^.

The *SHH* pathway is a central regulator of cerebellar development and its dysregulation has been implicated in the generation of a substantial fraction of the cerebellar medulloblastomas (MBs), which have been classified in four different molecular subgroups, *WNT*, *SHH*, group 3 and group 4^9,10^. The *SHH* group, characterized by inappropriate expression or aberration of *SHH* pathway genes, originates from committed granule neuron precursor cells (GNPCs) of the external granular layer of the cerebellum^11^. In normal physiology, the SHH signal produced by the adjacent Purkinje cells promotes GNPC proliferation and prevents differentiation; once the stimulus is terminated, cells exit the cell cycle and differentiate^12–14^. SHH inhibitors are considered promising agents for the development of targeted therapeutic strategies in MBs belonging to the *SHH* subgroup^15–20^.

The present study has been focussed on ZNF521 (also known as EHZF or Evi3)^21,22^, a 30 zinc finger transcription co-factor, an important regulator of the homeostasis of the immature hematopoietic cells^23–27^. ZNF521/Zfp521 has also been implicated in the control of neural development^28–32^, adipocyte differentiation^33–35^, maintenance of chondrocyte identity^36^ and bone formation^37,38^. Importantly, ZNF521 is highly expressed in the external granule layer of the cerebellum, where the GNPCs considered the cells-of-origin of MBs of the *SHH* subgroup are located^23^. Consistently, ZNF521 is particularly abundant in the *SHH* subtype of MB and has been shown to play a critical regulatory role in MB cells^39^.

These features prompted us to investigate if a functional cross-talk exists between ZNF521 and the *SHH* pathway. Our data, illustrated here, delineate a direct interaction of ZNF521 with the GLI1 and GLI2 transcription factors, which enhances the transcriptional activation of GLI target promoters through a mechanism that requires the presence of the N-terminal motif of ZNF521 and the recruitment of the nucleosome remodelling and HDAC (NuRD) complex.

## Results

### Correlation between the expression of ZNF521 and SHH target genes in MB

A set of 736 MB cases (R2 analysis platform, public database Tumour Medulloblastoma - Cavalli - 763 - rma_sketch - hugene11t^40^) was analysed for the expression of *ZNF521* in the different subgroups, where highest expression was found associated with the *SHH* subgroup, followed by the *WNT* subgroup and then group 4, with lowest expression in group 3 (Fig. S1A).

**Fig. 1:**
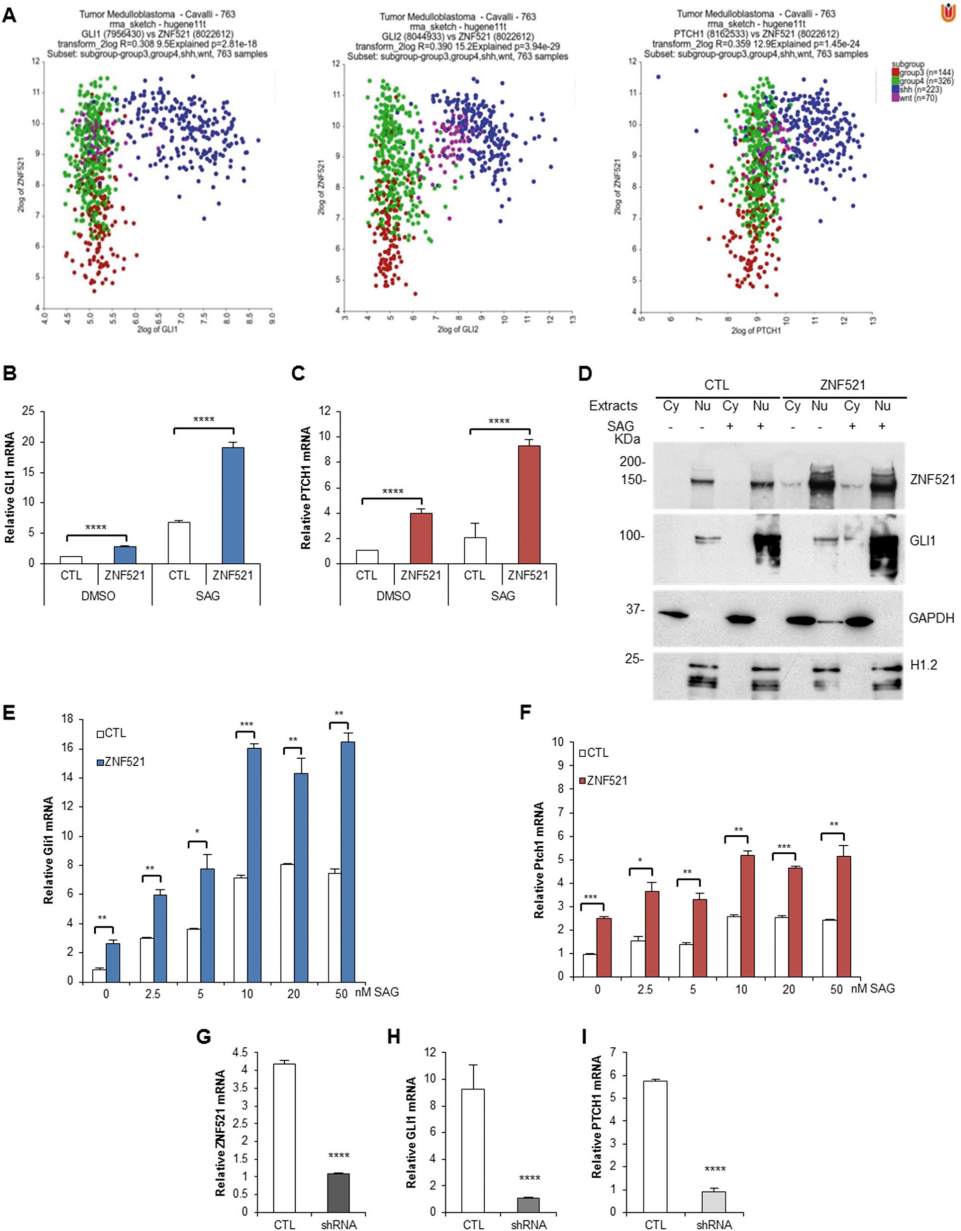
ZNF521 expression is associated with the SHH subgroup of MBs and promotes the activation of SHH pathway. **a** Association of ZNF521 with *SHH* pathway genes in subgroups of MBs. Co-expression (2 log XY plots) of *ZNF521* (8022612 reporter probe) associated with either *GLI1* (7956430 reporter probe), *GLI2* (8044933 reporter probe) or *PTCH1* (8162533 reporter probe). The different MB subgroups are indicated as WNT (pink dots), SHH (blue dots), group 3 (red dots) and group 4 (green dots). **b–h** Enforced overexpression of ZNF521 activates the *SHH* pathway. DAOY cells transduced with either *ZNF521* or FUIGW control vector (CTL) were stimulated with 200 nM SAG, the *SHH* agonist, for 48 h or without (DMSO). RT-qPCR analysis shows the increase in *GLI1* (**b**) and *PTCH1* (**c**) mRNA with *ZNF521* transduction and an additional increase with SAG. **d** These cells once transduced with *ZNF521* showed an increase in GLI1 protein predominantly in the nucleus, which was overexpressed when the cells are treated with 200 nM SAG. The cytosolic (Cy) and nuclear (Nu) extracts were controlled by the enrichment of GAPDH and histone H1.2, respectively. NIH3T3 transduced with *ZNF521* and incubated for 48 h with 0–50 nM SAG were analysed by RT-qPCR for *Gli1* (**e**) and *Ptch1* (**d**) mRNA. UW228 MB cells were silenced for ZNF521 using a lentiviral shRNA vector and analysed for *ZNF521* (**g**), *GLI1* (**h**) and *PTCH1* (**i**) mRNA expression by RT-qPCR. *p < 0.05, **p < 0.01, ***p < 0.001, ****p < 0.0001

In addition, an analysis was carried out to establish whether a correlation exists between the expression of *ZNF521* and that of individual components of the *SHH* pathway, including *GLI1*, *GLI2* and *PTCH1*. To this end, the messenger RNA (mRNA) levels of *GLI1*, *GLI2* and *PTCH1* were plotted against those of *ZNF521*. The scatter profile XY plot shows that the expression of *GLI1*, *GLI2* and *PTCH1* is particularly associated with the presence of high amounts of *ZNF521* transcript (Fig. 1a). There is an overall positive correlation (Fig. S1B) among *ZNF521* and the 763 clinical cases expressing *GLI1*, *GLI2* and *PTCH1*. However, when individual groups are considered, a negative correlation emerges (Fig. S1C). It is interesting to note that high expression of *ZNF521*, although found predominantly in cases of MB with expression of the *SHH* pathway, is also high in *WNT* cases (Fig. 1a, Fig. S1D). The *WNT* genes were also tested for a direct correlation with *ZNF521* in the MB cases and it was found that only *WNT5A* and *LEF1* had a significant correlation, whereas no association was found with other *WNT* genes (Fig. S3).

### Enforced overexpression of ZNF521 stimulates the expression of SHH target genes and phenocopies SHH signalling

In the light of the abundance of *ZNF521* mRNA in *SHH* MB and the association between its expression and that of *SHH* pathway mediators, depicted in Fig. 1 and Fig. S1, we sought to further characterize the possible existence of a cooperative cross-talk between ZNF521 and SHH signalling. As shown in Fig. 1b, c, lentiviral-mediated enforced expression of ZNF521 in DAOY cells enhanced by approximately two-fold the expression of the HH target genes, *GLI1* and *PTCH1*. Moreover, the presence of ZNF521 reinforced the *GLI1* and *PTCH1* up-regulation upon treatment with the SMO agonist, SAG^41^. Additional analyses of cytoplasmic and nuclear extracts confirmed that ZNF521 induced a small increase of nuclear GLI1 protein, which is enhanced once stimulated with SAG (Fig. 1d). The same trend was also observed in NIH3T3 cells in which *ZNF521*-overexpressing cells displayed *Gli1* and *Ptch1* mRNA up-regulation, which became more prominent upon SAG treatment in a dose-dependent manner (Fig. 1e, f).

The UW228 MB cell line was used to test for the role of endogenous ZNF521 in the *SHH* pathway. These cells express high amounts of ZNF521 and it was previously shown that once ZNF521 was silenced by lentiviral transduction of short hairpin RNA (shRNA), they displayed reduced growth, colony formation and migration^39^. Here it is found that once endogenous ZNF521 was silenced there was a marked decrease in both of the *SHH* pathway targets, *GLI1* and *PTCH1* mRNA (Fig. 1g–i).

**Fig. 2:**
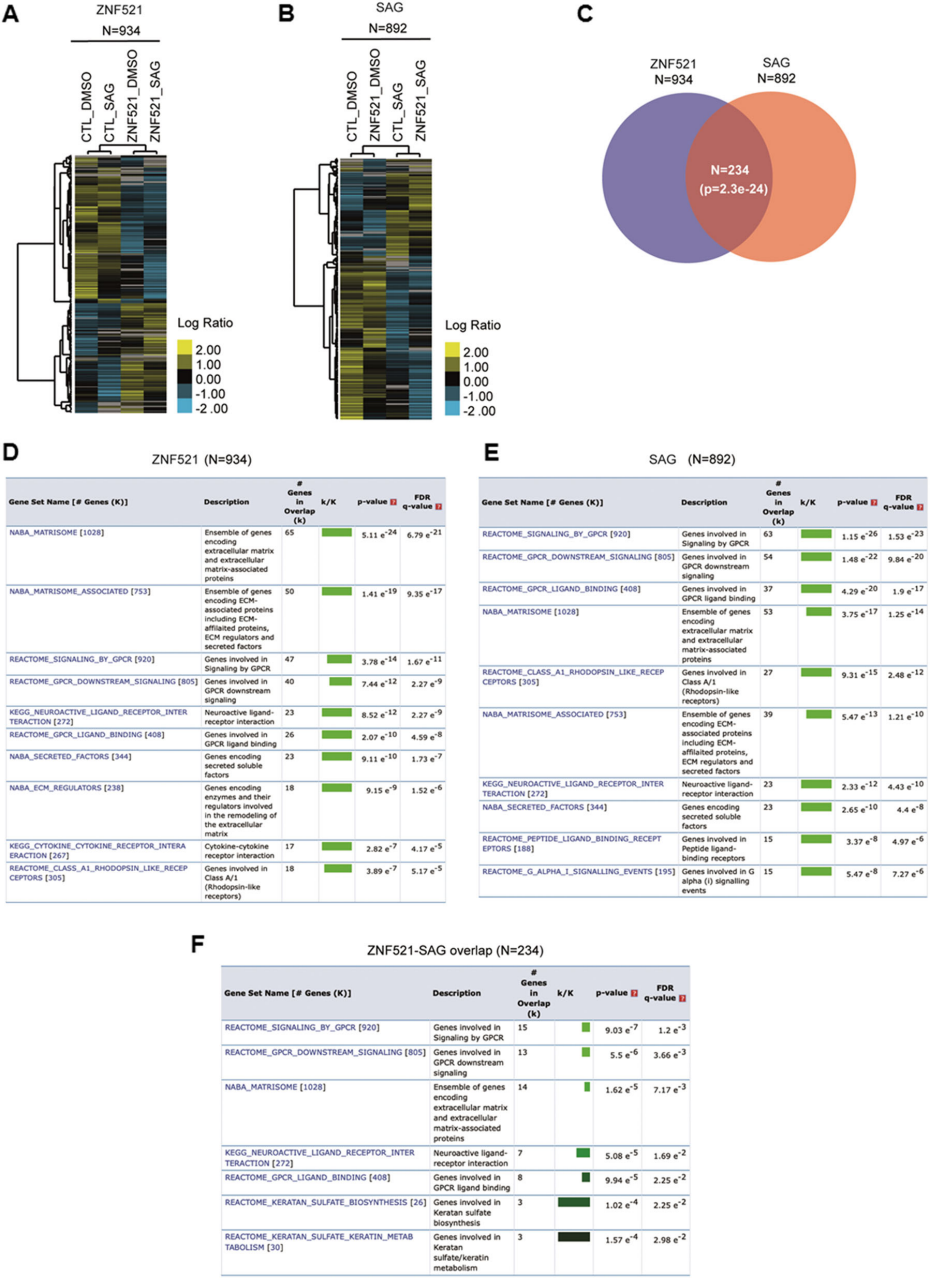
Gene expression profile analysis of DAOY cells. **a** Hierarchical clustering analysis of 934 genes differentially expressed (*p* value ≤ 0.05) in ZNF521-overexpressing cells versus control cells (CTL). **b** Hierarchical clustering analysis of 892 genes differentially expressed (*p* value ≤ 0.05) in SAG-treated cells versus untreated control cells (DMSO). Scale bars, the log 2 ratio of expression of mean centred genes. **c** Venn diagram using the 934 and 892 gene sets. A total of 234 genes were found overlapping. *P* *value* was calculated using the hypergeometric distribution test. **d–f** The top 10 overlapping gene sets (K) representing MSigDB canonical pathways (see also Methods) are shown for the 934, 892 or 234 genes (k). Colour bar shading from light green to black, where lighter colours indicate more significant “false discovery rate” (FDR) *q* *values* (<0.05) and black indicates less significant FDR *q* values (≥0.05)

We next analysed how ZNF521 modulate *HH* signalling and the overall transcriptome of DAOY cells. Gene expression profile analysis by RNA-sequencing (RNA-Seq) of DAOY cells revealed that the ectopic expression of ZNF521 modulates a total of 934 genes (*p* ≤ 0.05) with respect to control cells (Fig. 2a; Supplementary Table 1). On the other hand, by treating DAOY cells with SAG, a total of 892 genes (*p* ≤ 0.05) resulted significantly modulated when the SHH signalling is activated, regardless of the presence of ZNF521 with respect to the control treatment group (Fig. 2b; Supp. Table 2). Interestingly, we found 234 genes (*p* = 2.3e^−24^) overlapping between the 934 and 892 gene sets (Fig. 2c; Supplementary Table 3). Strikingly, when we analysed the overrepresentation of canonical pathways among these sets of genes (i.e. the 934, the 892, and the 234), we found that the top-scoring pathways in terms of significance (false discovery rate (FDR) *q* value ≤ 0.05) were related to SHH mechanisms, such as extracellular matrix remodelling, G protein-coupled receptor signalling and downstream signalling (Fig. 2d–f). Taken together, these results showed that ZNF521 expression modulation largely impacts the transcriptional profile of genes involved in SHH signalling.

### ZNF521 interacts with Gli1 and Gli2 proteins

**Fig. 3:**
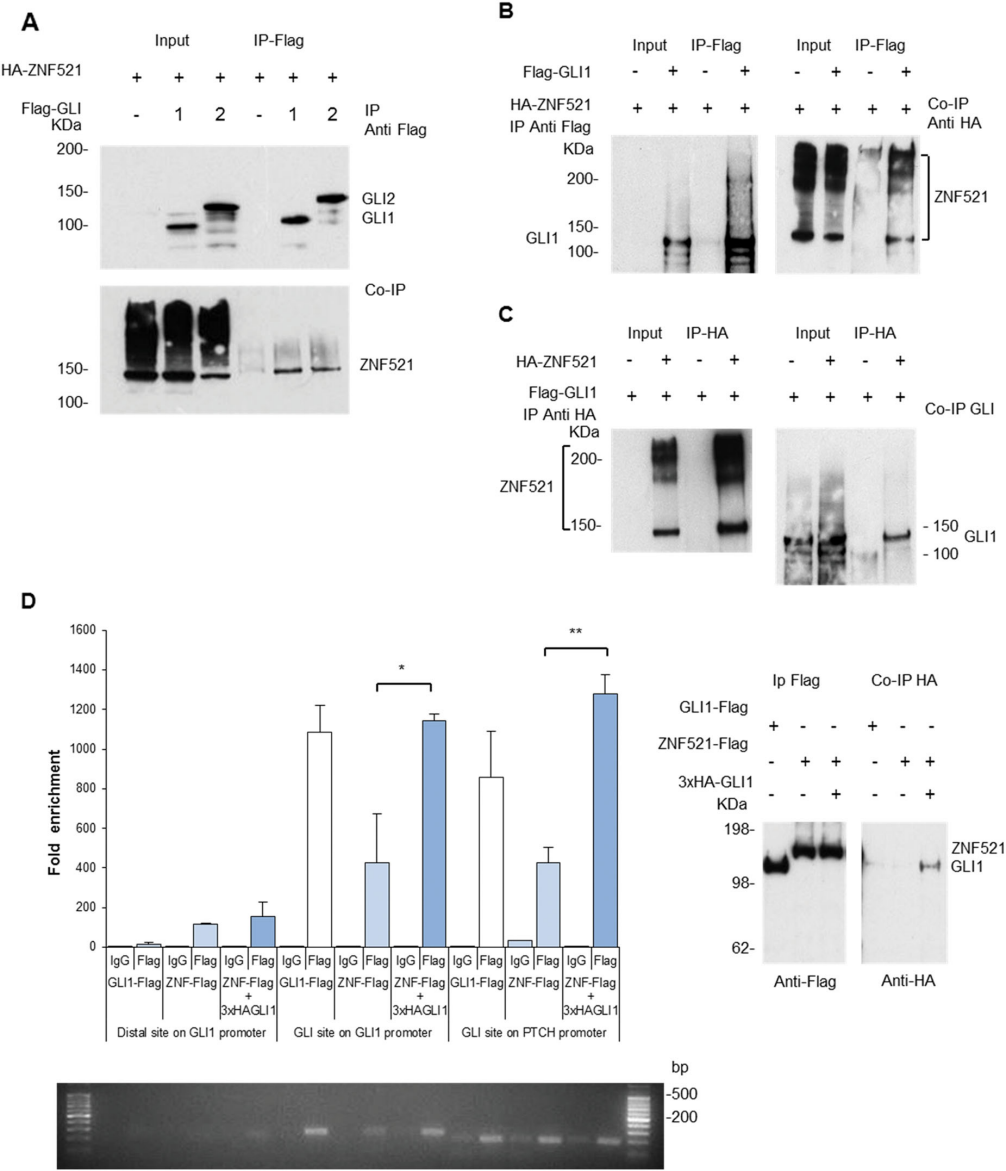
Interaction of ZNF521 and GLI1/ GLI2 demonstrated by Co-IP. **a** IP of Flag-GLI1 or Flag-GLI2 results in the Co-IP of HA-ZNF521. **b** IP of Flag-GLI1 results in the Co-IP of HA-ZNF521. **c** IP of HA-ZNF521 results in the Co-IP of Flag-GLI1. **d** ChIP demonstrates the pull down of *ZNF521* with the *GLI1* and *PTCH* promoter regions containing GBS, which was enhanced in the presence of additional GLI1. qPCR analysis of regions of the *GLI1* and *PTCH* promoters with GBS, pulled down either directly by Flag-*GLI1* or by 3xFlag-*ZNF521* alone, or by the combination of 3xFlag-*ZNF521* together with 3xHA-*GLI1*. Fold enrichment of amplified products is shown compared to the non-specific control in the absence of Flag antibody (IgG control). A distal region of the *GLI1* promoter was also amplified, which did not cover the GBS and acts as an internal control. PCR products were analysed by agarose gels and the proteins present in the IPs (ChIP) and co-immunoprecipitates (Co-IP) were analysed by Western blotting. *p < 0.05, **p < 0.01

To test whether a physical interaction occurs between ZNF521 and mediators of the *SHH* pathway, we performed co-immunoprecipitation (Co-IP) assays in HEK293T cells. Following co-transfection of cDNAs encoding tagged ZNF521, GLI1 and GLI2, ZNF521 co-immunoprecipitated with Flag-tagged GLI1 or GLI2 (Fig. 3a). In complementary Co-IP experiments, Flag-tagged GLI1 pulled down HA-ZNF521 (Fig. 3b), and conversely HA-ZNF521 co-precipitated Flag-GLI1 (Fig. 3c).

We then performed chromatin immunoprecipitation (ChIP) assays to establish whether the *GLI*/*ZNF521* interaction occurred at the chromatin level. Cells were transfected with either *GLI1*-Flag or *ZNF521*-Flag or with a combination of *ZNF521*-Flag and 3xHA-*GLI1* and sheared DNA pulled down with anti-Flag antibody or a control immunoglobulin G (IgG). The presence of the GLI1 binding sites (GBSs) was identified by amplifying across the sites with specific primers. Additional primers that amplified a distal region (2 kb) from the GBSs on the *GLI1* promoter, which gave a specific internal control, were also used.

These assays showed that both GLI1 (as expected) and ZNF521 were each pulled down in correspondence to the promoter regions containing consensus GBSs^42,43^. This was found for both *GLI1* and *PTCH1* promoters (Fig. 3d), but not (significantly) in the distal region of the *GLI1* promoter where no GBSs are present. Importantly, when ZNF521 (Flag-ZNF521) was directly pulled down, by anti-Flag antibody, in the presence of transfected GLI1 (3xHA-GLI1) (Fig. 3d Western blot, where 3xHA-GLI1 is seen as a Co-IP), there was an enrichment in the recovery of amplified fragments associated with the GBSs in these promoters compared to those found when Flag-ZNF521 was transfected alone (Fig. 3d, quantified by quantitative PCR (qPCR) and visualized by agarose gel). This indicates that ZNF521 together with GLI1 has a greater interaction with the *GLI1* and *PTCH1* promoter regions and is likely to enhance the interaction of GLI1 with its target promoters.

These data show for the first time a novel interaction between ZNF521 and the *SHH* principal effectors GLI proteins, which takes place at the chromatin level on the *GLI1* and *PTCH1* promoters.

### ZNF521 enhances the transcriptional activity of GLI1 and GLI2

**Fig. 4:**
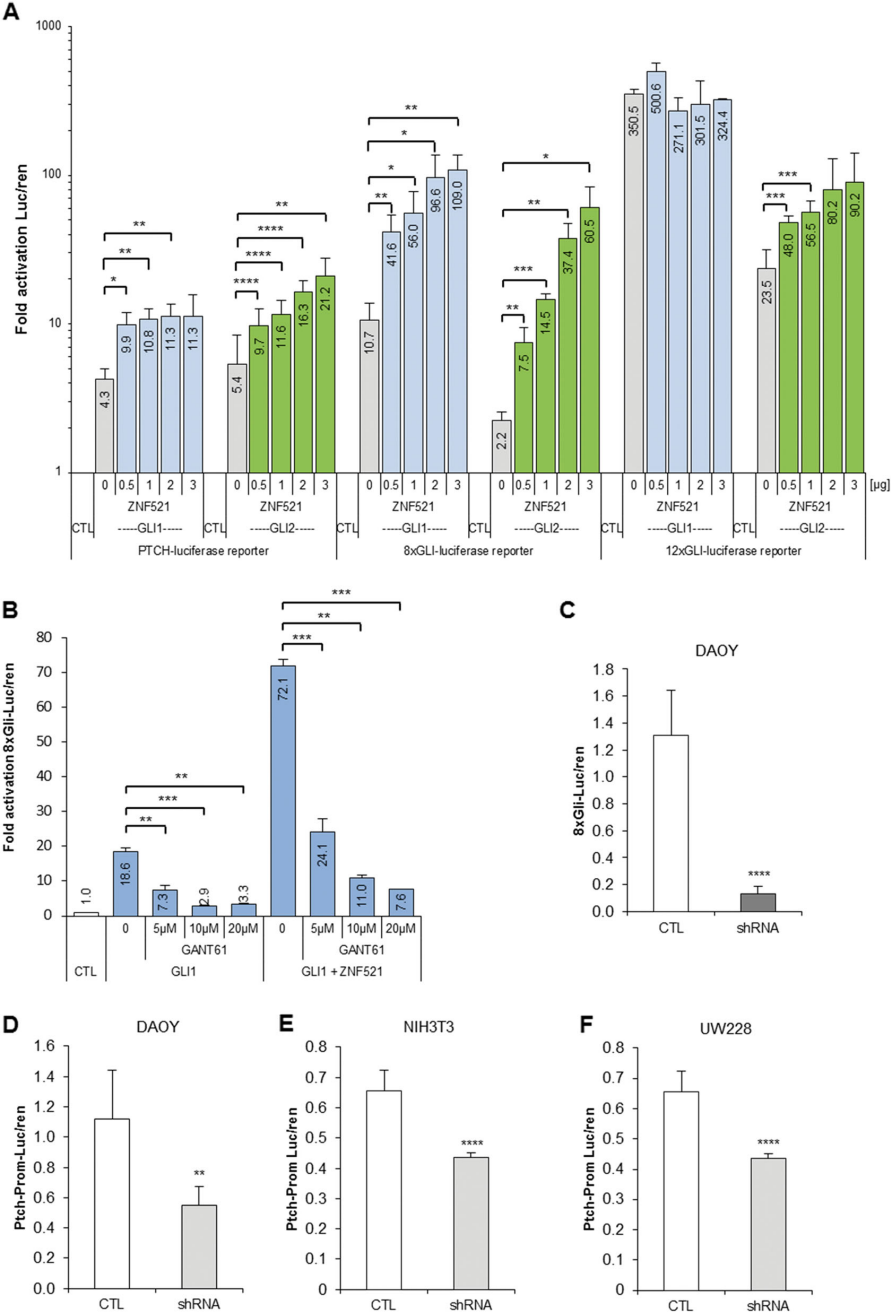
ZNF521 acts with GLI proteins to co-transactivate *GLI*-responsive reporter constructs. **a** The *GLI-*responsive reporters: *PTCH* promoter luciferase, 8x*Gli* (GBS) luciferase and the 12x*GLI-*(GBS) luciferase were transfected in HEK293T cells with constant amounts of either *GLI1* or *GLI2* alone or together with increasing amounts (0–3 μg/well) of *ZNF521* plasmid. The luciferase values were expressed as a ratio of the co-transfected Renilla activity (luc/ren). The results are expressed in a log scale as a fold activation in the absence of GLI1 or GLI2. **b** Transactivation of the 8x *Gli* reporter by GLI1 alone as well as with ZNF521 can be inhibited by 5–20 µM of the GLI1 inhibitor GANT61. **c–f** The cell line DAOY silenced for ZNF521 with shRNA had a reduced ability to transactivate the 8x*Gli-*luciferase construct (**c**). DAOY (**d**), NIH3T3 (**e**), and UW228 (**f**) cells silenced for ZNF521 with shRNA had a reduced ability to transactivate the *PTCH* promoter*-*luciferase construct. *p < 0.05, **p < 0.01, ***p < 0.001, ****p < 0.0001

To assess the contribution of ZNF521 in modulating the activity of GLI factors, transactivation assays were performed using reporter constructs containing GBSs fused to the cDNA of firefly luciferase (luc). To this end, cells were co-transfected with a reporter construct and expression vectors carrying the cDNAs for *ZNF521*, *GLI1* or *GLI2* as indicated in Fig. 4a. The *PTCH*-luc construct contains a 4.3 kb fragment of the *PTCH1* promoter region with one GBS at −704/−696 bp upstream of the transcription start site, while the engineered constructs 8x*Gli*-luc and 12x*GLI*-luc have multiple GBSs (Fig. S2). As expected (Fig. 4a), both *GLI1* and *GLI2* alone (grey bars) activated all the reporter constructs. Instead, the co-expression of ZNF521 with GLI proteins (GLI1, blue and GLI2, green bars) significantly enhanced the transactivation, almost invariably in a ZNF521 dose-dependent fashion.

This phenomenon was particularly striking with 8x*Gli*-luc, where ZNF521 expression resulted in a super-activation up to 10–30-fold greater than that induced by GLI1 or GLI2 alone. Instead, in the case of 12x*GLI*-luc, owing to the high responsiveness of this construct to GLI1 alone (350.5-fold activation), the additional effect of ZNF521 was masked by the already maximal activation level. Instead, the transactivation of 12x*GLI*-luc by GLI2 alone was relatively modest (23-fold activation) and the effect of ZNF521 resulted in a substantial up-regulation (up to 90-fold greater than that obtained by GLI2 alone).

The super-induction experiments described above were repeated in the presence of the GLI inhibitor GANT61, which specifically inhibits GLI1 binding to target promoters, thereby abrogating transactivation^44,45^. GANT61 treatment significantly inhibited, in a dose-dependent manner, 8x*Gli*-luc transactivation by GLI1 alone and also, to a large extent, the super-activation by the combination of GLI1 and ZNF521 (Fig. 4b).

Cell lines (DAOY, NIH3T3 and UW228) silenced for ZNF521 and transfected with 8x*Gli*-luc or the PTCH promoter luc reporter, which have a basal level of activity, were found to has a reduced transcriptional activation, indicating that the reduction of endogenous ZNF521 in these cells, which also express *SHH* pathway components, has attenuated the response (Fig. 4c–f).

These results delineate the existence of a cooperative action between ZNF521 and GLI factors in the transcriptional activation of GLI-binding regulatory sequences, which is sensitive to the GLI antagonist GANT61.

### The ZNF521-GLI1 cooperation requires the recruitment of the HDAC-NuRD complex by ZNF521

**Fig. 5:**
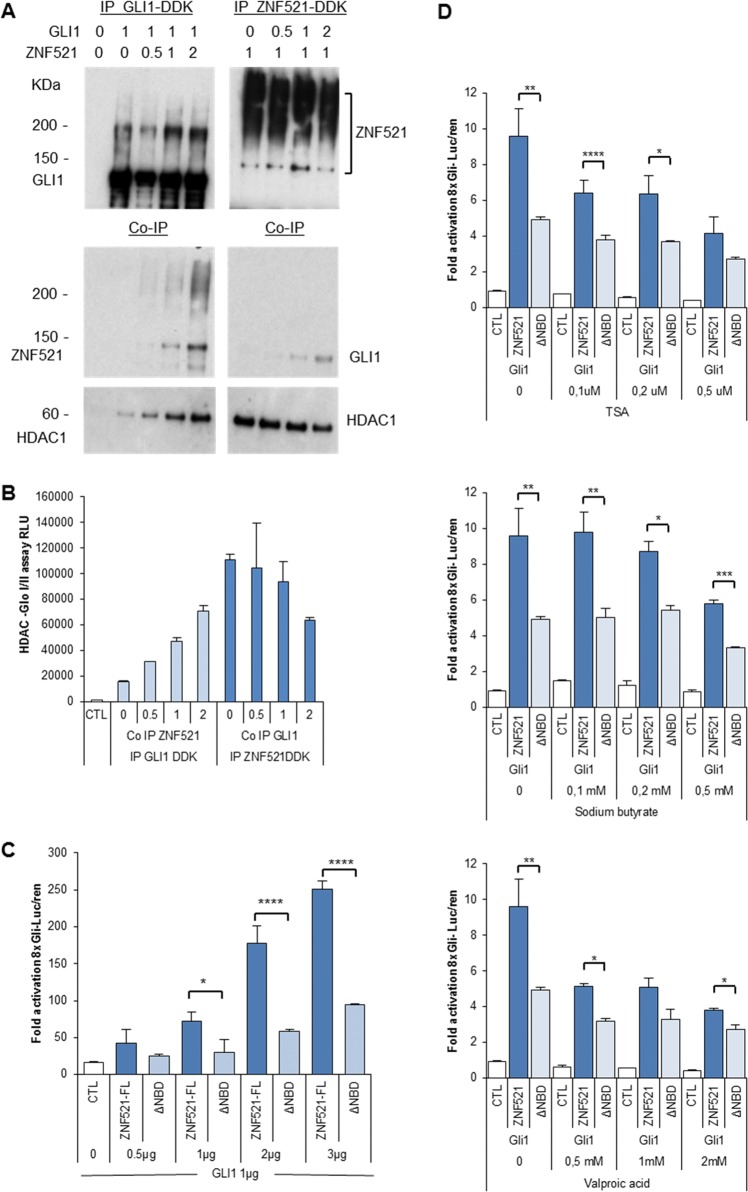
The activity of the combination of ZNF521 and GLI1 is promoted by the NuRD-interacting N-terminal motif in ZNF521. **a** Cells (HEK293T) were transfected either with *GLI1*-DDK (Flag) and increasing amounts of HA-*ZNF521* (top left panel) or with *ZNF521*-DDK (Flag) and increasing amounts of 3xHA-*GLI1* (top right panel). The IPs pulled down with anti-Flag-protein G complex were analysed with rabbit anti-DDK (Flag) antibody for GLI1 and ZNF521 and the Co-IPs with anti-ZNF521 or with anti-GLI1 antibodies, as well as for the presence of Co-IP HDAC1. **b** An aliquot of the IP beads were incubated with the 3xFlag peptide to release bound proteins and assayed with the HDAC-Glo™ I/II substrate and developer. **c** Cells were all transfected with GLI1, either with control vector (CTL) or with increasing concentrations of full-length (FL) ZNF521 or a construct lacking the first 12 amino acids (ZNF521-ΔNBD) and the 8x*Gli*-luciferase reporter. **d** Cells were transfected with GLI1 either with control vector (CTL) or together with ZNF521 or ZNF521-ΔNBD (used as control for HDAC inhibitors), and after 24 h, HDAC inhibitors, TSA, NaBt or VPA were added. The reporter activity was measured at 48 h and calculated as fold activation of the 8x*GLI*-luc. *p < 0.05, **p < 0.01, ***p < 0.001, ****p < 0.0001

ZNF521 possesses a 12-amino-acid-long motif at its N-terminal end^21–23,46–48^, which is needed to bind the NuRD complex, and is required for the activity of ZNF521 in MB cells^39^. To assess the relevance of this interaction in the cooperation between ZNF521 and *HH* signalling, we performed experiments in which increasing amounts of expression vector carrying the cDNA for HA-*ZNF521* were co-transfected with a constant amount of *GLI1*-DDK-Flag or, vice versa, increasing amounts of cDNA for 3xHA-*GLI1* were co-transfected with a constant amount of *ZNF521*-DDK-Flag (Fig. 5a). The extracts obtained were then subjected to IP with an anti-DDK-Flag antibody (Fig. 5a, top panel). The precipitates were analysed with antibodies to ZNF521, GLI1 or histone deacetylase 1 (HDAC1) (Fig. 5a, middle and bottom panels). As expected, ZNF521 was found associated with GLI1, and, reciprocally, GLI1 co-precipitated with ZNF521. As shown in Fig. 5a (lane 2), IP of GLI1 was accompanied by detectable co-IP of endogenous HDAC1, even in the absence of transfected ZNF521. However, in the presence of increasing amounts of transfected ZNF521, the co-precipitation of HDAC1 was increased proportionally. Instead, when ZNF521 was immunoprecipitated the amounts of HDAC1 that were pulled down were higher than that co-precipitated with GLI1, and when additional GLI1 was co-transfected, no increase in HDAC1 co-precipitation was observed. These data were confirmed by the measurement of HDACI/II activity in the precipitates (Fig. 5b), and indicate that the interaction between HDAC1 (or HDAC1-containing complexes, such as NuRD) and ZNF521 is preferential compared to that with GLI1.

In additional experiments, we tested whether the binding of ZNF521 to the NuRD complex is required for the ZNF521-GLI transcriptional co-operational activity. These assays demonstrated that co-transfection of *GLI1* (Fig. 5c) or *GLI2* (Fig. S2D) together with a deletion mutant of ZNF521 lacking the NuRD-binding domain (ΔNBD)—which is able to bind GLI1 (Fig. S2E) but unable to recruit components of the NuRD complex^23,24,46^—resulted in a lower enhancement of the 8x*Gli*-luc reporter activity than that achieved by full-length ZNF521. Furthermore, in a complementary set of experiments, treatment of transfected cells with three distinct HDAC inhibitors (trichostatin A (TSA); sodium butyrate (NaBt); valproic acid (VPA)) resulted in an aberration of the cooperative effect between ZNF521-FL and GLI1 (Fig. 5d) or GLI2 (Fig. S2F) on the 8x*Gli*-luc reporter, such that once the maximum amount of HDAC inhibitor was used, the difference between the full length and the construct lacking the NuRD-binding motif was minimized. Taken together, the data described above indicate that the association between ZNF521 and the HDAC-NuRD complex takes part in the transcriptional synergism between ZNF521 and the *HH* pathway effectors.

**Fig. 6:**
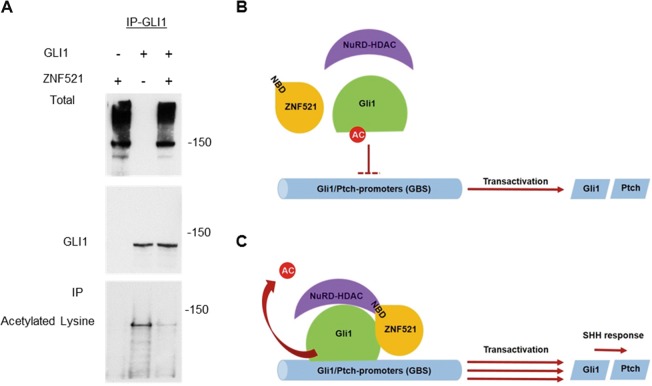
Activation of GLI1 is promoted by ZNF521 throught deacetylation of GLI1. **a** The presence of ZNF521 results in a reduced acetylation of GLI1. Cells were transfected either with *GLI1*-DDK (Flag) and 3xHA-*ZNF521* or with both together and IP was performed for GLI1 pulldown with anti-Flag-M2 agarose beads. Total input is shown for ZNF521 and IPs were hybridized with anti-GLI1 or with an anti-acetyl-lysine antibody. **b**, **c** Hypothesis for the mechanism of action for ZNF521 collaborating with GLI1 together with HDAC1-NuRD complex resulting in an increased transactivation of GLI-responsive genes. Diagrams for the action of GLI1 on the GLI1 target promoters in the absence (**a**) or presence (**b**) of ZNF521

It is known that post-translational modifications of GLI proteins contribute to the fine control of the *HH* pathway. Specifically, it has been documented that deacetylation of GLI factors by HDAC1 induces an increase in their transcriptional activity by facilitating their access to the chromatin^49–51^ and enhancing transactivation of target genes. To test whether the interaction with ZNF521 modifies the GLI1 acetylation status, *GLI1*-DDK-Flag and *ZNF521* were co-transfected and GLI1 was precipitated with anti-DDK-Flag antibody. Western blot analyses with an anti-acetylated lysine antibody showed a lower level of the acetylation of GLI1 in the presence of ZNF521 (Fig. 6a). This is likely to account for the increase in its activity induced by ZNF521.

## Discussion

ZNF521 displays features compatible with a function as a transcriptional regulator, with widely acknowledged roles in stem cells of diverse organs and tissues^23–30^. A notable feature being, a short amino acid sequence located at its N-terminal end, which is shared with a family of transcriptional regulators (friend of GATA-1/2, SALL1–4, BCL11a/b and Zfp423) and has been shown to recruit the NuRD complex^21,23,46,47,52–54^.

In the present approach, regarding neural neoplasias, ZNF521 was shown to be a regulator of immature MB cells, where its enforced expression stimulates growth, clonogenicity, migration ability and tumorigenicity, and this action requires the integrity of the NuRD-binding motif^39^. Among the MB subgroups, particularly high expression of ZNF521 is present in the subgroup characterized by dysregulation of the *SHH* pathway. Additional analyses of a larger cohort of patients, conducted in the framework of the present study, revealed a striking positive association between levels of *ZNF521* transcript and the expression of *SHH* pathway components, *GLI1*, *GLI2* and *PTCH*. It was observed that high expression of *ZNF521* was also detected in the *WNT* subgroup. However, when *WNT* genes were examined for correlations with *ZNF521* in the MB cases, only two genes *WNT5A* and *LEF1* showed a significant *R* value (Fig. S3); thus, *ZNF521* was not further examined in the context of having a role in the *WNT* pathway.

*ZNF521* being present at high levels in all SHH subgroup samples, it is likely to be crucial for this pathway, considering that low levels of ZNF521 are not found in this SHH subgroup. The functional and expression data for the *SHH* pathway^39^ encouraged us to explore the possibility that ZNF521 could physically and/or functionally interact with mediators of the SHH signalling. These results indicate that: (i) ZNF521 binds to GLI1 and GLI2; (ii) this interaction occurs in regulatory regions of SHH target genes that contain GLI-binding consensus sequences; (iii) enforced expression of ZNF521 in DAOY MB and NIH3T3 cells enhances the expression of SHH target genes both in the absence and in the presence of SAG; (iv) conversely, silencing of endogenous ZNF521 reduced GLI1 and PTCH1 mRNA; (v) RNA-Seq data showed that ZNF521 widely impacts the SHH signalling by modulating a large set of genes, whose expression is also regulated by the addition of SAG; (vi) ZNF521 synergistically cooperates with GLI1 and GLI2 in the transcriptional activation of SHH-responsive elements; and (vii) this synergism is more evident in the presence of the N-terminal NuRD-binding motif of ZNF521.

The *SHH* pathway is subjected to fine control, and the activity of GLI1 and GLI2 is known to be post-translationally modified by ubiquitination^50,55^ and sumoylation^56^ resulting in degradation. GLI1 can instead be activated by phosphorylation by the tyrosine kinase HCK, which disrupts the interaction with its inhibitor SUFU at the primary cilium^57^. An additional regulatory mechanism is mediated by acetylation/deacetylation of the GLI factors. Specifically, it has been determined that deacetylation of lysine 518 by HDAC1 is critical for the activation of GLI1^50^ and that the HDAC1/2 inhibitor, vismodegib, could inhibit tumour growth in a mouse MB model^51^.

Consistent with the notion that the HDAC-rich NuRD complex is necessary for the transcriptional cooperation between ZNF521 and GLI, three HDAC class I inhibitors were able to counteract the super-induction of the GLI reporter by full-length ZNF521. This evidence underlies the importance of the interaction between ZNF521 and HDAC1-NuRD in the enhancement of GLI transactivation. It also fits well both with our previous finding that the NBD is essential for the activity of ZNF521 in MB, and with those of Coni et al.^49,51^ and Canettieri et al.^50^ showing that deacetylation of conserved lysines in GLI1 and GLI2 augments the GLI transcriptional activity by permitting easier access to chromatin. This is further supported by our experiments in vitro, in which the co-expression of GLI1 and ZNF521 resulted in a significant deacetylation of GLI1 (Fig. 6a) and is exemplified in Fig. 6b as a mechanism whereby GLI activity is enhanced by ZNF521 through deacetylation dependent on NuRD-associated HDAC1.

The classification of MB into subgroups with distinct molecular, demographic and clinical characteristics has offered the possibility to determine prognostic differences between the groups. The current therapeutic strategies in MB treatment include surgical resection, cranium–spinal irradiation and adjuvant chemotherapy. The cure rates of average and high-risk patients are fairly high (85 and 70%, respectively)^58^; however, they are associated with serious treatment-induced morbidity. Inter-tumoral heterogeneity has been detected within the four different subgroups, such that a further classification identified a total of 12 MB subtypes^40^, which may permit an increased level of stratification by providing more specific prognostic information and thus allowing a greater degree of “therapeutic tailoring” to minimize treatment-induced damage. Molecular biomarkers associated with specific phenotypes of these tumours are potentially important in identifying and categorizing specific types of MBs. High expression of ZNF521 is found not only in the *SHH* group but also in the *WNT* group and in a substantial fraction of cases in group 4. ZNF521 expression could thus help define a novel sub-category classification. In this context, and in the light of the fact that activation of the *HH* pathway is critical for the maintenance of the stem cell compartment in tumours derived from dysregulation of different signalling mechanisms (ref. ^3^ and references therein), the NuRD-dependent synergism between ZNF521 and GLI might represent a valuable biomarker for the identification of patients with MB—and possibly other SHH-driven malignancies—who may potentially benefit from a combination of SHH and HDAC inhibitors.

## Materials and methods

### Cell lines and culture conditions

HEK293T, DAOY, NIH3T3 and UW228 cells were cultured in Dulbecco’s modified Eagle’s medium supplemented with 10% foetal bovine serum, 50 U of penicillin and 50 μg of streptomycin/mL, at 37 °C in 5% CO_2_. All tissue culture reagents were from Life Technologies. For stimulation of the *SHH* pathway, SAG SMO ligand (ALX-270–426, ENZO Life Sciences, Italy) was solubilized in dimethyl sulfoxide and used at 2.5–200 nM for 48 h, after the cells had been starved for 24 h.

### Plasmids and lentiviral vectors

The *ZNF521* lentiviral vectors *UBC* promoter-transgene-IRES-*EGFP* (FUIGW), FUIGW-flag-*ZNF521* and FUIGW-flag-*ZNF521ΔNBD* (lacking the first 12 amino acids that constitute the NuRD-binding motif) were used^24,39^ as well as TrueORF-Gold human NM_015461.2 pCMV6-*ZNF521*-Myc-DDK (Origene) and the mouse cDNA clones pCMV-3xHA-*Zfp521*^37^, Image Clone pCMV-Sport6-*Zfp521* (*Evi3*) NM_145492.4 was sub-cloned in the lentiviral vector FUIGW as well as the corresponding *Zfp521ΔNBD*. Human and mouse h*ZNF521*/m*Zfp521* are 97% identical in protein sequence and both or either constructs were used in the experiments and are indicated as *ZNF521*. shRNA lentiviral vectors for specific silencing of ZNF521 were as described in ref. ^39^.

*GLI1* and *GLI2* was transfected using the pCDNA-Flag-*GLI1*, pCDNA-3xHA-*GLI1* and pCDNA-Flag-*GLI2*, as well as TrueORF-Gold human NM_005269 pCMV6-*GLI1*-Myc-DDK (Origene) plasmids. Promoter reporter assays were performed with *PTCH*-luc (4.3 b promoter fragment)^59^, 8x*Gli*-luc, 12x*GLI*-luc (Cellogenetics) and transfections were normalized with the control pRL-TK Renilla plasmid (Promega).

### Transfection and transduction of cell lines

Plasmids were transfected using polyethylenimine (PEI) (Polysciences) 1 µg/µl (3 µg for 1 µg plasmid DNA) or the calcium phosphate method in HEK293T cells using 10 μg plasmid/100 mm tissue culture plate. The medium was changed after 16 h and cells were harvested for IP or luc activity after 48 h. Cells were transduced^60^ with the control vector FUIGW or FUIGW-*ZNF521* using the lentiviral packaging plasmids pCMV-VSVG and pCMV-deltaR8-91 and were 70–80% positive for the transgene EGFP (enhanced green fluorescent protein) by fluorescence-activated cell sorting analysis and stably expressed the ZNF521 or mutant ΔNBD at similar high levels. Cells were sorted for EGFP and were over 90% positive for EGFP.

### Expression analysis by RT-qPCR

RNA was prepared using the TRIzol reagent (Life Technologies), quantified with the NanoDrop 2000/2000c Spectrophotometer (Thermo Fisher Scientific) and the quality was monitored using 1.5% agarose gels run in MOPS buffer, pH 7.1 (0.4 M MOPS (3-(*N* morpholino)propanesulfonic acid), 0.1 M NaAc, 20 mM EDTA), and 10% formaldehyde. cDNA was synthesized from 1 µg RNA using SuperScript III reverse transcriptase at 42 °C and 2.5 µM random hexamers (Life Technologies). Quantitative reverse transcription-PCR (RT-qPCR) reactions were carried out with the iQ™ SYBR^®^ Green Supermix (Bio-Rad) with the qPCR amplifier QuantStudio3 (Applied Biosystems). One cycle of 3 min at 95 °C was followed by 45 cycles of 10 s at 95 °C, 10 s at 60 °C and 10 s at 72 °C, finishing with a melting curve. Relative gene expression was determined using the comparative threshold cycle Ct method, normalizing for housekeeping genes (*GAPDH* and *UBC*) such that the average the expression ratio was calculated as 2^−ddCt^. Primers used in this study were designed to have high stringency for qPCR and span exon–intron sites and the sequences were as follows (5′–3′): h-*GLI1* (F) ACAGCCAGTGTCCTCGACTT, (R) ATAGGGGCCTGACTGGAGAT; h-*PTCH* (F) CTTCGCTCTGGAGCAGATTT, (R) CAGGACATTAGCACCTTCT; h-*GAPDH* (F) CACCATCTTCCAGGAGCGAG, (R) TCACGCCACAGTTTCCCGGA; h-*UBC* (F) ATTTGGGTCGCGGTTCTTG, (R) TGCCTTGACATTCTCGATGGT; m-*Gli1* (F) ACCCACTCCAATGAGAAGCC, (R) CAGTTTGAGACCCCGAGACC; m-*Ptch* (F) TACCTCAACGGCCTACGAGA, (R) GCTGTCAGAAAGGCCAAAGC; m-*Gapdh* (F) TGACGTGCCGCCTGGAGAA, (R) AGTGTAGCCCAAGATGCCCTTCAG; m-*Ubc* (F) GCCCAGTGTTACCACCAAGA, (R) CCCATCACACCCAAGAACA.

### Transcriptome sequencing and analysis

Poly-A-enriched strand-specific ribosomal RNA-depleted libraries were generated with the Illumina TruSeq Stranded Total RNA Library Prep Gold, according to the manufacturer’s instructions. Libraries were sequenced by Illumina HiSeq2000 resulting in paired 50 nt reads. Fastq files were aligned to the hg38 genome assembly using STAR^61^. STAR was also used to quantify gene expression for each gene. STAR gene counts were normalized applying the median of ratios method implemented in DESeq2 R package^62^. Briefly, the normalization process implies different steps: (i) for each gene, a pseudo-reference sample is created and is equal to the geometric mean across all samples; (ii) for every gene in a sample and for each sample, the ratios sample/ref are calculated; (iii) the median value of all ratios for a given sample is taken as the normalization factor (size factor) for that sample; (iv) for each gene in each sample the normalized count values is calculated dividing each raw count value by the sample’s normalization factor. BRB-ArrayTools (v4.6; https://brb.nci.nih.gov/BRB-ArrayTools/) were used to perform statistical tests, briefly: (i) normalized count values were thresholded if the intensity at the minimum value was below 1; (ii) genes were excluded if <25% of expression data have at least a 1.5-fold change in either direction from gene’s median value; (iii) paired *t* test (with random variance model) were used to select features with a significant gene expression change (*p* value < 0.05) in the selected conditions. Clustering analysis was performed by using Cluster 3.0 and Java TreeView (http://bonsai.hgc.jp/~mdehoon/software/cluster/software.htm). The uncentred correlation and centroid linkage were used to aggregate genes and conditions. Pathways overrepresentation analysis was performed by using the Molecular Signature Database v6.2 (MSIgDB)^63^.

### Nuclear and cytoplasmic extracts

DAOY cells treated with or without 200 nM SAG were processed for nuclear and cytoplasmic extracts^64^. Cells were scraped, re-suspended in hypotonic lysis buffer (10 mM HEPES, pH 7.9, 10 mM KCl, 0.1 mM EDTA, protease inhibitors (P8849, Sigma) and phosphatase inhibitor cocktails 2 and 3 (P0044, P5726, Sigma) and incubated on ice for 20 min. After the addition of 0.25% Igepal-630 (NP40), samples were centrifuged at 3600 r.p.m for 5 min and supernatants containing the cytoplasmic extracts were recovered. Nuclear pellets were re-suspended in 20 mM HEPES, pH 7.9, 0.4 M NaCl, 1 mM EDTA with protease and phosphatase inhibitors. After three cycles of vortex and ice, samples were centrifuged at 12,000 r.p.m. for 20 min and the supernatants containing the nuclear extracts were collected. Proteins (50 μg) were denatured, reduced, separated on 4–12% NuPAGE Novex Bis-Tris gradient polyacrylamide gels (Life Technologies) and blotted onto nitrocellulose membranes. Membranes were quenched with 5% blotto (Bio-Rad), ZNF521 was detected with rabbit anti-ZNF521 (EHZF S15 sc-84808, Santa Cruz, Biotechnology; predicted molecular weight (MW): 148 kDa) at 1:2000, GLI1 with rabbit anti-GLI1 (C68H3, Cell Signalling; predicted MW: 118 kDa) at 1:5000, for nuclear extracts with rabbit anti-H1.2 at 1:10,000 (ab-4086, Abcam; predicted MW: 21.3 kDa) and cytoplasmic extracts with anti-GAPDH at 1:1000 (sc-166574, Santa Cruz Biotechnology; predicted MW: 36 kDa). Secondary rabbit and mouse horse radish peroxidase (HRP) antibodies were detected by the ImmunoCruz Western blotting luminal reagent (sc-2004, sc-2005, Santa Cruz, Biotechnology) and exposure to auto-radiographic film (GE Healthcare).

### Co-IP interaction assays

Cells, HEK293T cultured to 60–70% confluence, were transfected using PEI and were used for IP after 48 h. After washing with 1× phosphate-buffered saline (PBS), cells were pelleted at 1000 r.p.m. for 5 min at 4 °C and re-suspended in 1 ml of IP buffer (50 mM Tris/HCl, pH 7.5, 250 mM NaCl, 0.1% Triton X-100, 0.1 nM ZnCl_2_) supplemented with protease and phosphatase inhibitor cocktails 2 and 3. Cell lysates were sonicated on ice for three times for 10 s at 100% amplitude (UP50H ultrasonic processor Hielscher, Ultrasound technology) and centrifuged twice at 13,000 r.p.m. for 20 min at 4 °C to remove debris.

IP was performed with 10 μg monoclonal anti-Flag-M2 (F3165, Sigma-Aldrich) and 20 μl Protein G Sepharose (P3296, Sigma-Aldrich). Cell lysates were added to the beads and incubated overnight at 4 °C with rotation. After four washes with IP buffer and then with PBS, bound proteins were released at 95 °C for 5 min in NuPAGE^®^ LDS Sample Buffer plus reducing agent and loaded onto a NuPAGE Novex Bis-Tris 4–12% gel. Antibodies used for the detection of immunoprecipitated proteins were anti-Flag-M2-HRP (A8592), DYKDDDDK Tag (Flag) rabbit antibody (#2368 Cell Signalling), rabbit anti-HA (ab9110, Abcam) and rabbit anti-GLI1 (C68H3, Cell Signalling).

HDACI/II activity assays were performed on Flag-M2-agarose IPs after releasing proteins with 3xFlag peptide at 200 μg/ml for 30 min at 4 °C, serial dilutions were made for the assays and extracts incubated with the HDAC-Glo™ I/II substrate and developer (G6420, Promega) for luminescence reading with the GloMax Explorer luminometer in white 96-well plates. The anti-acetyl lysine antibody (ab21623, Abcam) was used to control the degree of acetylation in IPs of GLI1-myc-DDK in the presence of ZNF521.

### Chromatin immunoprecipitation

ChIP was performed using *ZNF521*-myc-DDK- and/or *GLI1*-Flag-transfected 293T cells; 2 × 10^6^ cells in 500 μl PBS were cross-linked with 1% formaldehyde for 8 min and blocked with 1 mM glycine for 5 min, then washed twice with PBS and finally the cell pellets were frozen on dry ice. Cells were extracted in 200 μl of 20 mM HEPES, pH 7.9, 25% glycerol, 420 mM NaCl, 1.5 mM MgCl_2_, 0.2 mM EDTA and 1 nM ZnCl_2_, and then nuclei were recovered after centrifugation at 13,000 × *g* for 10 min. Nuclei were lysed and sonicated for 40 cycles of high voltage 30 s on/30 s off in a Bioruptor bath sonicator (Diagenode) at 4 °C in 300 μl of 50 mM Tris/HCl, pH 8.0, 1 mM EDTA, 150 mM NaCl, 0.2% SDS, 1% Triton X-100 and 1 nM ZnCl_2_. After centrifugation, it was verified that the sonication had resulted in fragments of 500–1000 base pairs (bp). Extracts were diluted with 1 ml of 50 mM Tris/HCl, pH 8.0, 1 mM EDTA, 150 mM NaCl, 0.1% Triton X-100 and 1 nM ZnCl_2_, and then centrifuged at 13,000 × *g* for 20 min at 4 °C. Soluble extracts were divided for specific IP with anti-Flag-M2 antibody and non-specific (control mouse IgG). Antibody complexes were recovered with Protein G Sepharose. After 16 h of mixing by rotation at 4 °C, the beads were washed three times with Triton buffer and twice with PBS and then de-cross-linked by incubation in 2.5 mM Tris/HCl, pH 6.8, 200 mM NaCl, 2% SDS and 10 mM dithiothreitol at 65 °C for 16 h. All buffers contained the protease inhibitor mix for His-tagged proteins from Sigma. DNA was extracted with phenol/chloroform, and precipitated with 0.3 M NaAc, pH 5.4, and 2.5 volumes of ethanol with 1 μg glycogen carrier. Pellets were washed with 70% ethanol and re-suspended in 30 μl H_2_O for qPCR analysis.

Primers used for amplification of the *GLI1* and *PTCH* promoters were: *GLI1* promoter distal ~2000 bp upstream, *GLI1* promoter proximal ~200 bp upstream covering the GBS, *PTCH* ∼700 bp upstream covering the proximal GBS, and were as follows (5′–3′): *GLI1* promoter distal (F) TAAGTGGGCTTTAGTGAGGGGCT, (R) TCTACGTCTCGAAGTTCTGGAGG; *GLI1* promoter proximal (F) CGTAAGCAGTATAGGGTCCCTCA, (R) ACCCGCGAGAAGCGCAAACTT; *PTCH* promoter proximal (F) GTATTGCTGCGAGAAGGTGG, (R) TTTCTGCGACGCGATTGGCTCG.

### Transactivation reporter assays

Cells were transfected with plasmids for GLI proteins and ZNF521 together with the reporter *PTCH*-luc, 8x*Gli*-luc or 12x*GLI*-luc normalizing with pRT-TK Renilla using the PEI transfecting reagent. After 40 h, cells were washed with PBS and total soluble extracts were prepared by freezing and thawing in 250 mM Tris/HCl, pH 7.5, containing protease inhibitors. Proteins were quantified using the Bio-Rad reagent and 10 μg was used for the Dual-Glo luc assay system (E2920, Promega) performed in white 96-well plates and detected using the GloMax Explorer Luminometer (Promega). Ratios of luc/Renilla luminescence were calculated and presented as fold activation for each reporter.

The GLI1 inhibitor GANT61 (S8075, Selleckchem) was prepared in ethanol and used at 20–100 nM added 4 h after transfection. HDAC class I inhibitors, TSA (T8552, Sigma-Aldrich) used at 0.1–0.5 μm in DMSO, NaBt (B5887, Sigma-Aldrich) used at 0.1–0.5 mM prepared in PBS and VPA (ALX-550-304 from Enzo Life Sciences) used at 0.5–2mM in DMSO were added 24 h after transfection before assaying at 48 h.

### Gene expression analysis of R2 platform database

The public data set of 763 tumour MB^40^ samples (Tumour Medulloblastoma - Cavalli - 763 - rma_sketch - hugene11t) (https://hgserver1.amc.nl/cgi-bin/r2/main.cgi) was interrogated for *ZNF521* (8022612) vs. *GLI1* (7956430), *GLI2* (8044993) or *PTCH* (8162533) reporter probes, as well as for *WNT* genes. For graphical representation and statistical comparison between the subgroups and subtypes, data were transferred into Excel and the GraphPad prism version 5.03 program for analysis (*t* test of unpaired two-tailed analysis)

### Statistical analysis

The Student’s *t* test assuming unequal variances between two samples was used to determine the significant differences. Groups were judged to differ significantly at *p* values lower than 0.05.

## Supplementary information

Supplemental Table 1-3

Figure S1

Figure S2

Figure S3. Correlation between ZNF521 and WNT pathway genes in all Mb subgroups

Supplementary figure legends
