# Supplementary figures and images for: The stem cell-associated transcription co-factor, ZNF521, interacts with GLI1 and GLI2 and enhances the activity of the Sonic hedgehog pathway
Source: Cell Death Dis. 2019 Sep 26;10(10):715. doi: 10.1038/s41419-019-1946-x (PMC6763495; doi:10.1038/s41419-019-1946-x)

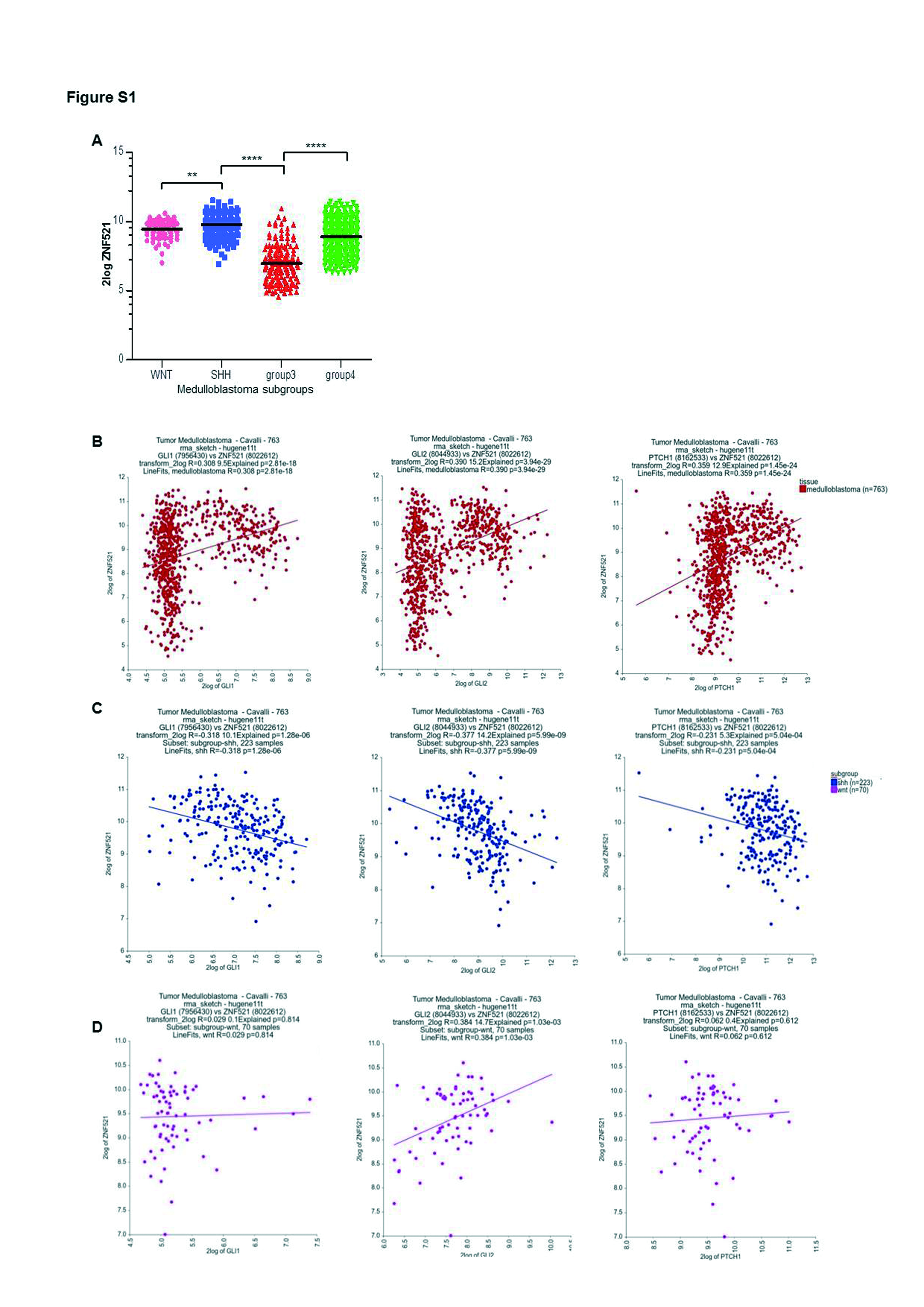

Supplement: Supplementary file 2 — Figure S1 [file 41419_2019_1946_MOESM2_ESM.tif]

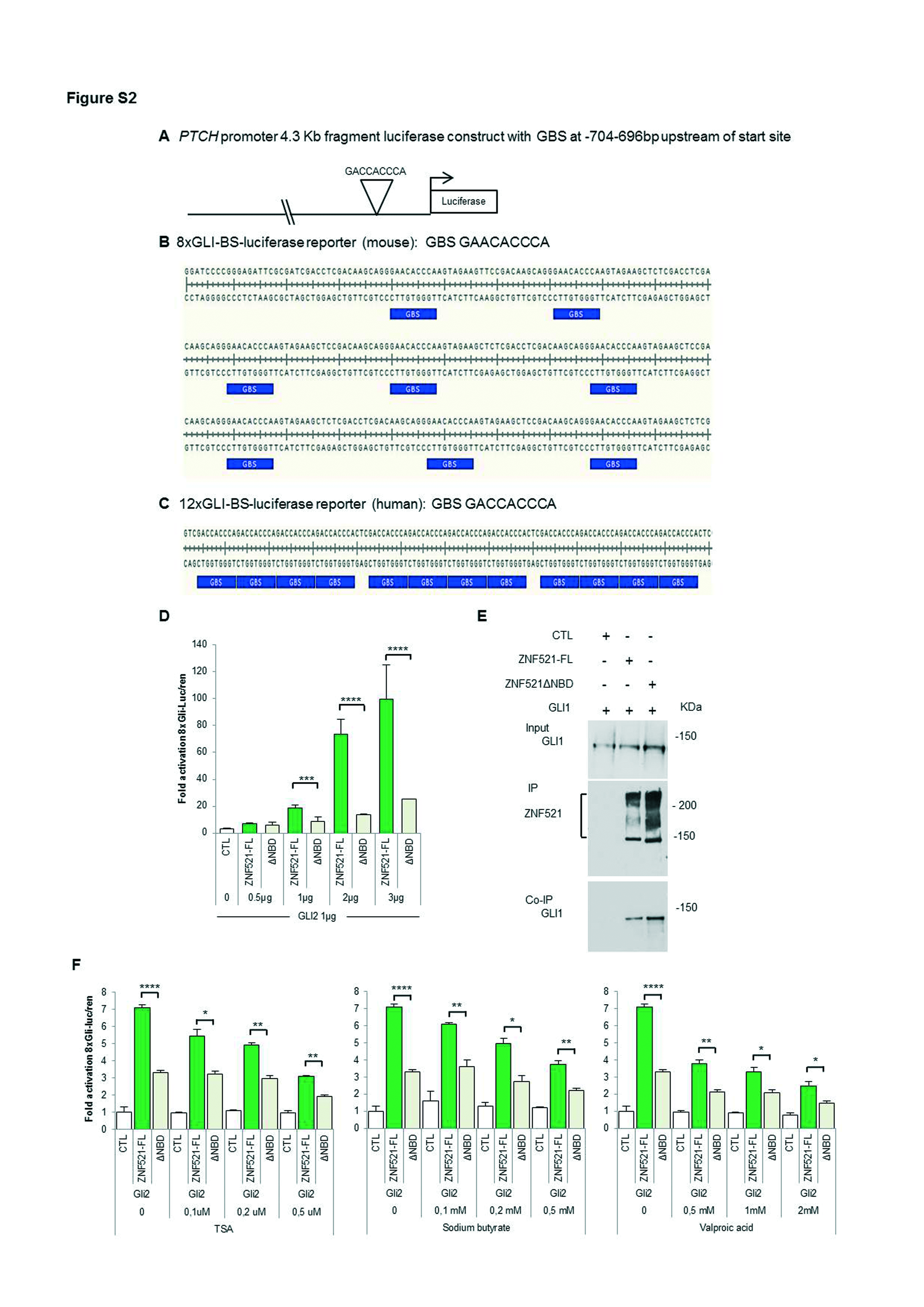

Supplement: Supplementary file 3 — Figure S2 [file 41419_2019_1946_MOESM3_ESM.tif]

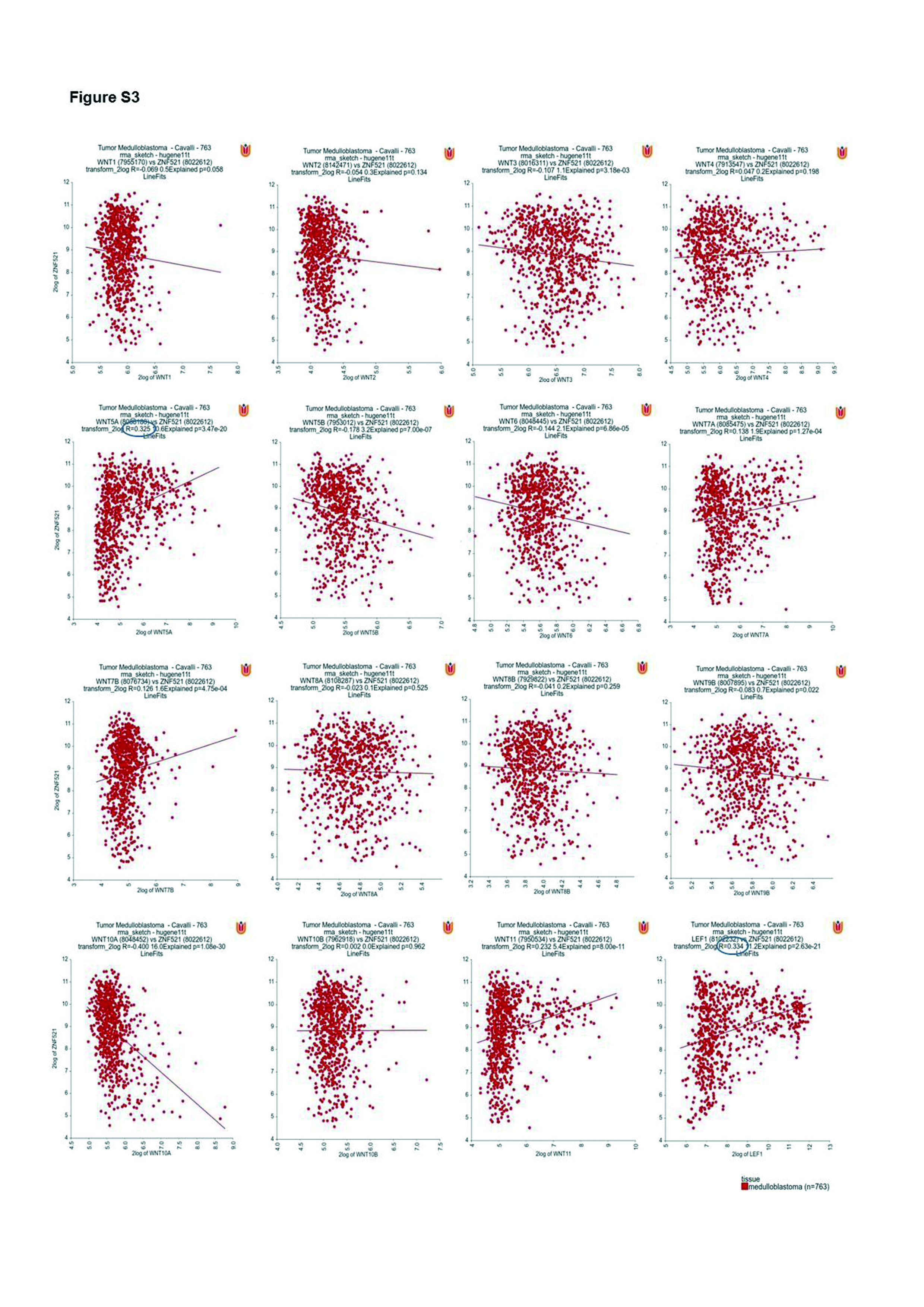

Supplement: Supplementary file 4 — Figure S3. Correlation between ZNF521 and WNT pathway genes in all Mb subgroups [file 41419_2019_1946_MOESM4_ESM.tif]
