# Supplementary material for: The stem cell-associated transcription co-factor, ZNF521, interacts with GLI1 and GLI2 and enhances the activity of the Sonic hedgehog pathway
Source: Cell Death Dis. 2019 Sep 26;10(10):715. doi: 10.1038/s41419-019-1946-x (PMC6763495; doi:10.1038/s41419-019-1946-x)
Supplement: Supplementary file 5 — Supplementary figure legends [file 41419_2019_1946_MOESM5_ESM.docx]

**Supplementary Table (1-3).** The list of genes and relative normalized reads counts are reported being modulation by ZNF521 (934 genes), and by SAG treatment (892 genes) and the overlapping 234 gene set. Columns are labeled based on the respective experimental conditions.

**Figure S1. ZNF521 expression in medulloblastoma subgroups.**

1. Relative ZNF521 mRNA levels (expression array database - Tumor Medulloblastoma - Cavalli - 763 - rma_sketch - hugene11t) are compared in MB patients in the different subgroups: WNT (70 samples), SHH (223 samples), group 3 (144 samples) and group 4 (326 samples). For graphical representation and statistical comparison between the subgroups data (T-test of unpaired 2 tailed analysis) were transferred into Excel and the GraphPad prism version 5.03 program.
2. Trend lines are shown for the correlation of ZNF521 with GLI1,GLI2 and PTCH1 in the 763 samples of MBs.
3. ZNF521 is plotted against either GLI1,GLI2 or PTCH1 within the SHH subgroup of the MBs

**D.** ZNF521 is plotted against either GLI1,GLI2 or PTCH1 within the WNT subgroup of the MBs.

**Figure S2.**

**A-C. Diagrams for the GLI responsive luciferase reporters.** The *PTCH* promoter contains one consensus *GLI* binding site (GBS) at position -704-696bp (**A**). The 8x*Gli*-luc reporter plasmid contains eight GBSs interspersed by 22bp (**B**). The 12x*GLI*-luc plasmid contains 12 GBS closely positioned, in 3 groups of 4 (**C**).

**D-F. The synergistic transactivation of ZNF521 and GLI2, is promoted by the NuRD interacting N-terminal motif on ZNF521.** Cells were all transfected with *GLI2*, either with control vector (CTL) or with increasing concentrations of full length (FL) *ZNF521* or a construct lacking the first 12 amino acids (*ZNF521-ΔNBD*) and the 8x*Gli*-luciferase reporter (**D**).Cells were all transfected with plasmids expressing 3xHA-*GLI1* together with control vector (lane 1), Flag-*ZNF521*-FL (lane 2) or Flag-*ZNF521-ΔNBD* (lane 3) and IP carried out with FlagM2 antibody for ZNF521. Co-IP GLI1 was detected with anti-GLI1 antibody (**E)**. Cells were transfected with GLI2, either with control vector (CTL) or together with ZNF521 or ZNF521-ΔNBD. After 24h HDAC inhibitors, TSA, NaBt or VPA were added and the reporter activity measured at 48h and calculated as a fold activation of the 8x*Gli*-luc (**F**).

**Figure S3**. **Correlation between** **ZNF521 and *WNT* pathway genes in the MB subgroups.**

High expression of *ZNF521*, was detected in the *WNT* subgroup (expression array database - Tumor Medulloblastoma - Cavalli - 763 - rma_sketch - hugene11t). When the *WNT* genes were examined for correlations with *ZNF521* in the MB cases, only two genes *WNT5A* and *LEF1* showed a significant positive correlation (R=0.325, p=3.47e-20 and R=0.334, p=2.63e-21).
